# Enzyme-Linked Aptamer Kits for Rapid, Visual, and Sensitive Determination of Lactoferrin in Dairy Products

**DOI:** 10.3390/foods11233763

**Published:** 2022-11-22

**Authors:** Fan Zhang, Hongxia Du, Linsen Li, Tengfei Li, Jing Wang, Zilei Chen, Mengmeng Yan, Chao Zhu, Feng Qu

**Affiliations:** 1 Institute of Quality Standard and Testing Technology for Agro-Products, Shandong Academy of Agricultural Sciences, Jinan 250100, China; 2 Shandong Provincial Key Laboratory Test Technology on Food Quality and Safety, Jinan 250100, China; 3 Key Laboratory of Molecular Medicine and Biotherapy, School of Life Science, Beijing Institute of Technology, Beijing 100081, China; 4 School of Life Sciences and Food Engineering, Hebei University of Engineering, Handan 056038, China; 5 School of Materials Science and Engineering, Hebei University of Engineering, Handan 056038, China

**Keywords:** aptamer, lactoferrin determination, enzyme-linked aptamer assay, colorimetric, capillary electrophoresis

## Abstract

Lactoferrin (Lf), as a popular nutritional fortification in dairy products, has the ability regulate the body’s immune system and function as a broad-spectrum antibacterial, which is of great significance to the growth and development of infants and children. Herein, an indirect competitive enzyme-linked aptamer assay (ELAA) kit was established for rapid, sensitive, and visual determination of Lf in dairy products. In the construction, the Lf aptamer was conjugated with horseradish peroxidase (HRP) as the recognition probe and aptamer complementary strand (cDNA) were anchored onto the microplate as the capture probe. The recognition probes were first mixed with a sample solution and specifically bound with the contained Lf, then added into the microplate in which the free recognition probes in the mixture were captured by the capture probe. After washing, the remaining complex of cDNA/Aptamer/HRP in the microplate was conducted with a chromogenic reaction through HRP, efficiently catalyzing the substrate 3, 3′, 5, 5′-tetramethylbenzidine (TMB), therefore the color shade would directly reflect Lf concentration. Under the optimization conditions, a good linear relationship (R^2^, 0.9901) was obtained in the wide range of 25–500 nM with the detection limit of 14.01 nM and a good specificity, as well as the reliable recoveries. Furthermore, the ELAA kits achieved the Lf determination with an accuracy of 79.71~116.99% in eleven samples, which consisted of three kinds of dairy products: including goat milk powder, cow milk powder, and nutrition drop. Moreover, the results were also validated by the high-performance capillary electrophoresis (HPCE) method. The ELAA kit provides a simple and convenient determination for Lf in dairy products, and it is highly expected to be commercialized.

## 1. Introduction

Lactoferrin (Lf) is an iron-binding glycoprotein with a molecular weight of about 80 kDa, which is abundant in mammalian milk and plays an important role in both innate immunity and the growth of neonates, infants and young children [1,2]. Until now, Lf has been extensively studied and is demonstrated to have a wealth of functions such as broad-spectrum antibacterial [3], antiviral infection [4], regulating the body’s iron balance [5], mediating the production of bone marrow cells [6], inhibiting human tumor cells [7], effectively treating diseases in synergy with various antibiotics and antifungal agents [8], etc. Due to its efficacy, Lf has been one of the fastest developing nutritional additives in recent years, and its products are increasingly used in the processing of dairy products, especially common in milk powder which is crucial for non-breastfed neonates and infants [9,10,11]. Hence, the sensitive detection for LF is of great significance in the fight against food adulteration and in support of market surveillance, and eventually for ensuring its function to be available in the organism. In China, the “Food Nutrition Fortification Use Standard (GB14880-2012)” stipulates that the maximum allowable use of Lf in infant formula is 1.0 g/kg, while few National Standards related to Lf determination in dairy products have been established.

The detection methods for Lf in dairy products are mainly classified as physicochemical techniques, biosensor assays, and immunoassays [12]. Among the physicochemical techniques, high-performance liquid chromatography (HPLC)-derived methods have been continuously and widely studied and applied, such as reverse-phase HPLC [13], size exclusion-HPLC [14], HPLC-fluorescence [15], and HPLC-tandem mass spectrometry (HPLC-MS/MS) [16]. Besides, electrophoresis methods, especially high-performance capillary electrophoresis (HPCE), are also attracting considerable attention with their advantages of high separation efficiency and fast analysis speed [17,18,19]. Although the physicochemical instrumental methods are generally the traditional methodology, some aspects involving inconvenient portability, the complicated pre-treatment, requirement of expensive instruments and professionals, sometimes negatively affect their application. Recently, biosensing technologies such as electrochemical sensors [20,21] and the surface plasmon resonance [22] method have been employed due to their simplicity, accuracy and sensitivity, and practicality. For example, Ebrahimi et al. [23] constructed an electrochemical sensor based on the Lf adsorption by the MOP self-assembled monolayer for Lf determination in colostrum samples with a low detection limit of about 65.2 nM. These methods still contained some shortcomings such as low throughput, poor reproducibility, and low environmental applicability, despite the biosensors being simple and having the sensitivity to determine Lf. The antibody-based immunoassays have been widely integrated with handy devices, such as enzyme-linked immunosorbent assay (ELISA) [24], microfluidic chip [25], and strips [26], but the process of antibody preparation requires animal immunization experiments and has a long development period. Given the diverse functions of Lf and the existing detection dilemma, it is of great significance to establish simple, convenient, and efficient methods for Lf determination.

Recently, Qu’s group obtained an aptamer against Lf using CE-SELEX with an equilibrium dissociation constant (K_D_) of 20.74 nM [27], which provided a basis for the establishment of an aptamer biosensor for Lf determination. An Aptamer is a short single-stranded nucleotide sequence that binds to a target molecule with high specificity and affinity, selected from a random oligonucleotide library by systematic evolution of ligands by exponential enrichment (SELEX) [28,29]. Known as “chemical antibodies”, aptamer has the advantages of simple preparation, stable properties, a wide range of targets, low immunogenicity, and easy modification compared with the antibody [30,31]. Currently, with the advancement of aptamer screening and synthesis methods, its downstream applications are becoming more and more extensive: such as medicine, life science, and bioanalytical science [32,33]. Among the numerous aptasensors, the horseradish peroxidase (HRP) enzyme-linked aptamer assay (ELAA) kit [34,35], similar to the well-established and approved antibody-based ELISA kit in the market, is one of the most anticipated applications because of its simplicity, rapidity, and visualization.

In this study, an indirect competitive ELAA kit was established for Lf determination in dairy products. The HRP-labeled aptamers were reacted sequentially with the measured samples and the aptamer’s complementary strand (cDNA) immobilized on the microplate to rapidly determine Lf content through the color change based on the enzyme-catalyzed substrate. This sequential reaction avoids excessive binding of the aptamers to cDNA, thus ensuring full-coverage recognition of Lf. The ELAA kit presented high sensitivity and stability in Lf determination, as well as good spiked recoveries, and was successfully applied to the determination of Lf in thirteen samples obtained from the market. In addition, the workflow of the ELAA kit could be accomplished through the convenient “mix-wash-detect” operation for an entire time within 60 min, which exhibited a certain potential as the rapid and efficient method for Lf determination in dairy products.

## 2. Experimental Sections

### 2.1. Reagents, Materials and Instruments

Lf standards (from bovine milk), bovine serum albumin (BSA), α-lactalbumin (α-la), β-lactoglobulin (β-lg), transferrin (Tf) were purchased from Sigma-Aldrich LLC (Beijing, China). Streptavidin (SA) and biotin-modified HRP were purchased from Aladdin Reagent (Shanghai, China). The 96-well plate was purchased from Thermo Fisher Scientific (Waltham MA, USA); single component 3, 3′, 5, 5′-tetramethylbenzidine (TMB), TE buffer, PBS buffer (pH 7.2) were purchased from Solarbio Biological Technology Company (Beijing, China). H_2_SO_4_ and Tween-20 were supported by Beijing Reagent Plant (Beijing, China). Polyethylene glycol dodecylether (Brij 35) was purchased from Macklin Inc. (Beijing, China). The Aptamer sequence of biotin-TGGTGCTGCCCCCCTAGTCTCCGGCTGCTTCTTGG and its complementary chain sequence of biotin-CCAAGAAGCAGCCGGAGACTAGGGGCAGCACCA were ordered and purified by Shanghai Sangon Biotechnology Co., Ltd. (Shanghai, China). The capillary was obtained by Sino Sumtech (Handan, Hebei, China).

The HPCE model is WooKing HPCE512 produced by Hanon Group (Jinan, China) and equipped with a UV detector (214 nm). The absorbance at 450 nm was measured by a Microplate reader (Multiskan™ FC, Thermo Fisher Scientific, Waltham MA, USA). The samples were prepared using the circular oscillator (MS 3 digital, IKA, Staufen, Germany) and high-speed refrigerated centrifuge (Neofuge 15R, Heal Force, ShangHai, China). The ultrapure water used throughout the experiments was purified by a Milli-Q system (Bedford MA, USA) and had a resistivity of 18.2 MΩ cm. Before use, all solutions were filtered through a 0.22 μm filter (Boston, MA, USA).

### 2.2. The Preparation of Capture Probe

First, SA was diluted in carbonate buffer (50 mM, pH 9.6) and 50 μL of SA solution was added into each well of the 96-well microplate and incubated for 1 h at room temperature which was controlled at about 25 °C by air conditioning. After that, the wells were poured and washed three times with PBS-T buffer (1×PBS buffer with 0.05% Tween-20) and patted dry. To reduce non-specific adsorption, the 96-well plate was blocked by adding 300 μL of BSA solution to the wells that were not completely coated with SA, and the wells were blocked for 1 h. Then, the wells were washed three times with PBS-T, each time retaining the washing solution for 1 min. As a result, the SA-modified microplate was obtained.

The biotin-modified cDNA chain was diluted with TE buffer (pH 8.0), and 50 μL solution was added to each well of the SA-modified microplate and incubated for 10 min. After that, the solution in the wells was poured out and washed three times with PBS-T buffer and patted dry. The prepared capture probe was eventually stored in a refrigerator at 4 °C for later use.

### 2.3. The Preparation of Recognition Probe

The SA-modified HRP (1 mg/mL) was diluted to the desired concentration using PBS (pH 7.2), and then was mixed with biotin-labeled aptamer at a ratio of 7:3. The mixture was added into a centrifuge tube and incubated for 30 min on a rocking bed. In the end, the prepared recognition probe was stored in a refrigerator at 4 °C for later use.

### 2.4. The Operation of ELAA Kit

The recognition probes were first mixed with the Lf samples in equal volumes for about 5 min at room temperature. Then, 100 μL of the mixture solution was loaded into each well of the microplate to incubate with a capture probe for about 30 min. The microplate was washed three times with PBS-T and patted dry. In subsequent steps, 100 μL of TMB solution was separately added into each well, and after 5 min 50 μL of 2 M H_2_SO_4_ solution was added to terminate the enzyme catalysis. The absorbances (A_450_) were scanned by a Premium reader, the relative A_450_ was used to calculate the Lf content, which was obtained by the formula of *(A_b_ − A_s_)/A_b_* where *A_b_* represented the A_450_ value of blank and *A_s_* represented the A_450_ value of the sample.

### 2.5. Evaluation of Selectivity and Stability

In the selectivity assay, the concentration of Lf protein was 300 nM, and the concentration of all other proteins was 3 µM. The stability assay was verified within 15 days after keeping the constant of capture probes and recognition probes. During the experiments, the temperature of 25 °C and the Lf concentration of 100 nM were kept constant. The assays were performed under optimal experimental conditions, and the final absorbance value at 450 nm was measured to evaluate the method specificity.

### 2.6. Treatment of Dairy Products

First, 0.05 g of powder or 0.05 mL of nutrition drops was dissolved in 1 mL of acetic acid solution (50 mM) and mixed well. The mixture was centrifuged at 8000 r/min for 10 min. Three layers of samples were obtained after centrifugation, from top to bottom, fat layer, clear liquid layer, and precipitation layer. The 200 μL of the clear liquid layer was aspirated with a syringe and subsequently filtered through a 0.22 μm cellulose acetate membrane to obtain the sample solution. The sample solution was analyzed with an ELAA kit.

### 2.7. HPCE Assay

The HPCE for Lf determination was referenced by Li et al. [18]. The bare capillary (50 μm i.d.) with a total length of 32.6 cm (effective length 20.3 cm) was rinsed with 1 M NaOH for 25 min, and water for 5 min at the first use. In the HPCE assay, the sequential rinses for capillary were required between each run for 3 min with 1 M NaOH, water, and running buffer solution, in which the composition was 40 mM NaH_2_PO_4_, 40 mM H_3_PO_4_, and 5 mM Brij 35. The sample was injected at 0.5 psi for 20 s or the required time. The detection wavelength was set to 214 nm. During the separation, the high voltage of 12 kV (the inlet as the anodes) and the temperature of 22 °C were maintained.

## 3. Results and Discussion

### 3.1. Working Principle of the ELAA Kit

**Figure 1:**
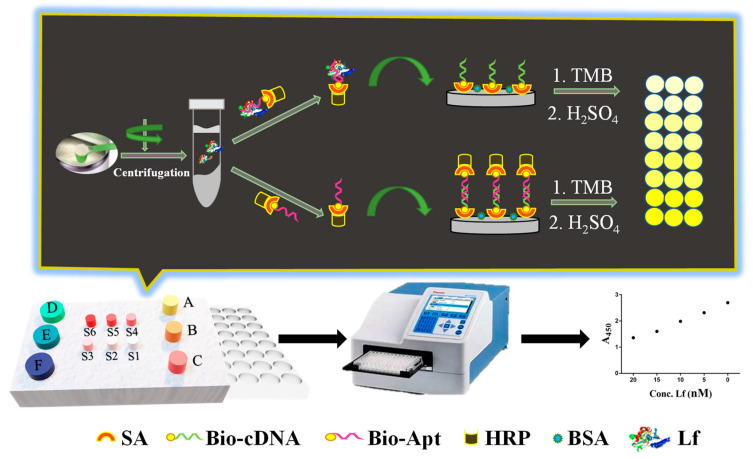
The construction and schematic of the ELAA kit for Lf determination.

The working principle of the ELAA kit was illustrated in Figure 1 as follows. Initially, the capture probe of the biotin-labeled cDNA was anchored onto the surface of both streptavidin (SA)-coated and BSA pre-blocked polystyrene microplate wells through the specific interaction between SA and biotin. The recognition probe was constructed through the conjugation between SA-modified HRP and biotin-labeled aptamer. In the absence of Lf in a sample, there is no binding event between the aptamer and Lf, therefore the recognition probe would subsequently hybridize with the capture probe through the base complementary pairing between the aptamer and cDNA. After washing, the rich complexes of aptamer/biotin-SA/HRP remained in the wells, in which numerous HRPs were available for the added reporters of TMB to be adequately catalyzed, yielding a sharp color change from pale to dark yellow. In the presence of Lf in the sample, the aptamer elements-affiliated recognition probe would first target Lf analytes due to their specific recognition and high-affinity binding, which thereby lead to a decrease in the number of unbound recognition probes. Correspondingly, fewer complexes of aptamer/biotin-SA/HRP remained and caused insufficient catalysis of the TMB along with the color change at different degrees, in which color shades were negatively correlated with Lf concentrations in the sample. The displayed ELAA kit was performed by employing a convenient “mix-wash-detect” workflow and a sequential reaction-based indirect competition that ensured full-coverage binding of Lf to achieve a robust determination, and reduced the feasibility of the accessibility to nonspecific binding sites on the surface. The results of the yellow shade were directly observed by the naked eye and the absorbance (A_450_) was scanned by a Premium reader, which enabled achieving semi- and quantitative determination, respectively.

### 3.2. Optimization of Experimental Conditions

**Figure 2:**
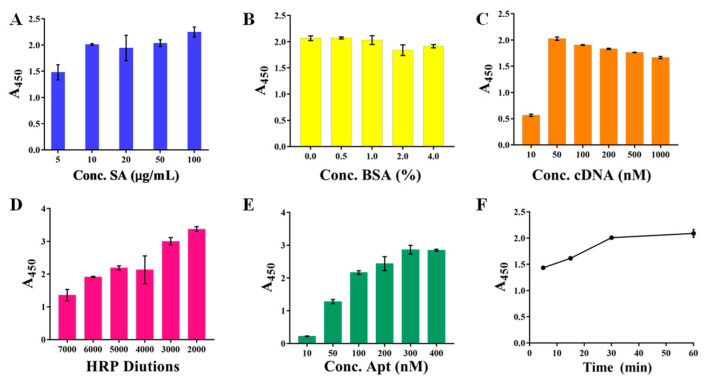
The optimizations of capture probe and recognition probe involving the concentrations (**A**) of SA, the concentrations (**B**) of BSA blocking solution, the concentrations (**C**) of the cDNA, the concentrations of SA-HRP (**D**) and biotin-Apt (**E**), as well as their incubation time (**F**).

The careful optimizations of a series of parameters were conducted. These optimized conditions ensured that the final color was relatively easy to distinguish and that the rapid and accurate determination of the Lf was fully guaranteed. First, three aspects in the constructions of the capture probe were optimized involving the concentrations of SA modified onto the bottoms of a microplate, the concentrations of BSA blocking solution, and the concentrations of the cDNA. Figure 2A displayed that the SA concentration reached 10 μg/mL, and the A_450_ value no longer increased with the increased concentration, but leveled off, which the concentration then ensured that the actual SA amount coated onto the microplate reached saturation, and was thus selected. Figure 2B showed that the A_450_ value of 0.5% BSA was fundamentally the same as that of unblocked, indicating that the microplate was coated successfully. In addition, the A_450_ value decreased as the BSA concentration increased, indicating that the high concentration of BSA would instead reduce the kit’s sensitivity. The 0.5% BSA was still selected to prevent non-specific adsorption despite its seeming inability. Similarly, the cDNA concentration of 50 nM (Figure 2C) presented a relatively high and stable A_450_ value and was selected. Particularly, the high cDNA concentration resulted in a slightly reduced A_450_ value, most likely due to the mismatched binding between cDNAs.

Subsequently, the constructions of the recognition probe were optimized, including the concentrations of SA-HRP and biotin-Apt, as well as their incubation time, which determined the amount of the bound Lf and the catalyzed TMB, thereby significantly affecting the kits analytical performance. Figure 2D showed that the A_450_ value increased with the increasing SA-HRP concentration, in which the high concentration at the dilution of 1:2000 and 1:3000 gave excessively high A_450_ values (>3.0) that reached the higher limit of the detector, whereas the dilution of 1:5000 presented a relatively suitable A_450_ value of 2.5 and its color was easy to distinguish with the naked eye, thereby employed in the following experiments. As anticipated, more biotin-Apt probes could provide more opportunities for accessibility of the cDNAs, resulting in the higher signal output of the A_450_ value (Figure 2E), and the concentration from the moderate part of 200 nM was selected. Figure 2F displayed the incubation time between SA-HRP and biotin-Apt mounted up to 30 min, the absorbance approached a relatively higher value, and in the next 30 min the increase was only 5.4%. Thus, we chose 30 min as the optimal incubation time.

**Figure 3:**
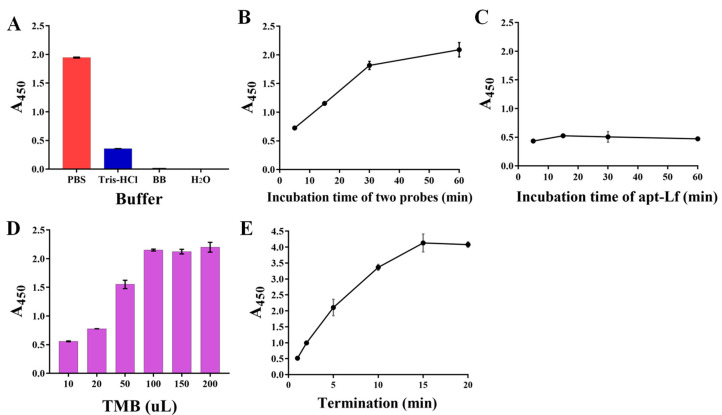
The optimizations of the procedures in detection operation involving the incubation buffer (**A**), the binding time of the recognition probes separately to subsequent capture probes (**B**) and to the Lf target (**C**), the added volume (**D**) of TMB, and the termination time (**E**).

Furthermore, the procedures in the detection operation were optimized, including the incubation buffer, the binding time of the recognition probes separately to the Lf target and to subsequent capture probes, the added volume of TMB, and the termination time. The results displayed that: the PBS buffer presented sufficient signal output whereas the other three buffers did not (Figure 3A); A_450_ increased obviously with the increasing two probes’ binding time from 0–30 min, whereas in the next 30 min the increase was only 5.4% (Figure 3B); the absorbance at the binding time of 5 min decreased to a relatively low value (Figure 3C) and then became plateau due to the high affinity between the aptamer and Lf protein; the TMB volume of 100 µL (Figure 3D) and the termination time of 5 min guarantee the relatively suitable absorbance (Figure 3E), whereas although a longer termination time could yield larger A_450_ values, the corresponding excessively dark color was not easily distinguished by the naked eye; thereby, these parameters were employed in the following experiments.

### 3.3. Performances of ELAA Kit

**Figure 4:**
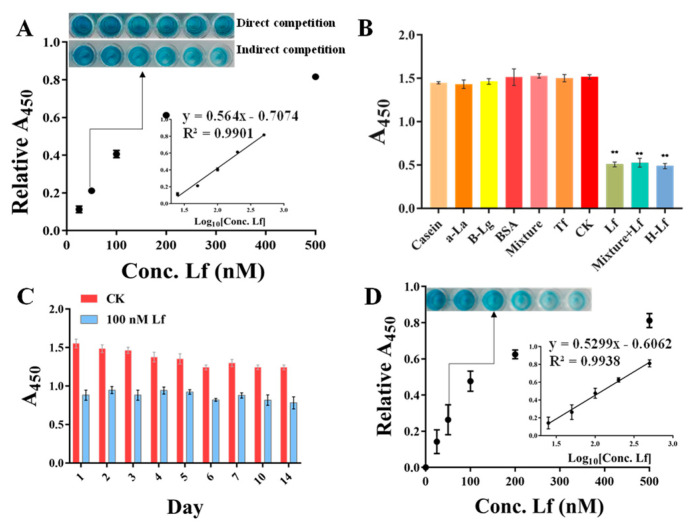
The performances of ELAA kit involve sensitivity (**A**), specificity (** Represents a significant difference, **B**), and stability (**C**), and the calibration curve and sensitivity in spiked matrix (**D**).

Under the optimization conditions, the sensitivity was evaluated using a standard Lf solution of different concentrations ranging from 25 to 500 nM. As shown in Figure 4A (insert picture), with the concentration of Lf increased, the blue color of the solution gradually becomes evidently lighter with the naked eye. However, the direct competition-based assay displayed no significant difference in color. A good calibration curve was fitted with a reliable correlation coefficient (R^2^) of 0.9901 based on the linear relationship between the relative A_450_ values (vertical coordinate) and the Lf concentration (horizontal coordinate). Error bars were obtained based on three parallel measurements at different concentrations. Meanwhile, the regression equation was obtained by y = 0.564x − 0.7074, along with a limit of detection (LOD) of 14.01 nM (1.12 mg/L) based on the 3σ (σ = α/k, α = 2.6345 is the standard deviation of the blank signal, k = 0.564 is the slope of the standard curve), whereas the direct competition-based ELAA showed a poor R^2^ of 0.7193 and a higher LOD of 347.14 nM. Therefore, it could be concluded that the indirect competition-based ELAA kit was capable of determining Lf quantitatively. Although the LOD was at an intermediate level compared to other reported ELISA methods (Table S1) [26,36,37,38], it fully meets China’s GB14880-2012 which shall not exceed 1.0 g/L, along with a convenience “mix-wash-detect” operation. In addition, these ELISA methods are based on the immunoreaction between Lf and its antibody, which are expensive reagents and complicated to prepare.

To verify the selectivity of the ELAA kit and prevent the occurrence of false positives, other proteins were evaluated simultaneously by the ELAA kit, including casein accounting for a large proportion in milk, and the main whey proteins of bovine serum albumin (BSA), α-lactalbumin (α-la), β-lactoglobulin (β-lg), and their mixture, as well as the blank samples as controls (CK). As shown in Figure 4B, the wells of only Lf and Lf-contained a mixture which presented similar A_450_ nm values, whereas the blank sample and other individual proteins, as well as their mixture and even with 10-fold concentration, provided higher and similar absorbance, indicating that the ELAA kit had a good selectivity. In addition, the specificity was also evaluated using other proteins of human lactoferrin (H-Lf) that belong to different genera, and Transferrin (Tf) that also belongs to the transferrin family. Figure 4B depicts that the ELAA kit presented a similar recognition to H-Lf, perhaps due to their similar structures, as well as a high specificity in transferrin recognition. These results all indicated that the ELAA can be used in the specific recognition and determination of Lf.

To assess the reproducibility of the ELAA kit, we performed three groups of experiments including the intra-batch assay, intra-day assay, and inter-day assay. The A_450_ values were measured using three Lf standards of 60, 80, and 100 nM. The coefficient of variation (CV) is a relative measure of dispersion and is often used as an important indicator for reproducibility. A higher CV value means a more significant detection error and a lower value means a more stable result. As listed in Table 1, the calculated average CVs of intra-day, inter-day, and intra-batch assays for the ELAA kit were respectively 0.01–0.07, demonstrating the reproducibility of the ELAA kit based on the low-value. Moreover, the stability of the ELAA kit was verified within 14 days by utilizing the same lot of kits. Figure 4C showed that A_450_ values were not significantly changed with a good relative standard deviation (RSD) of 5.72%, indicating that the ELAA kit has good stability and can be used to prepare kits for long-term use for a certain period of time.

### 3.4. Calibration Curve and Sensitivity in Spiked Matrix

**Figure 5:**
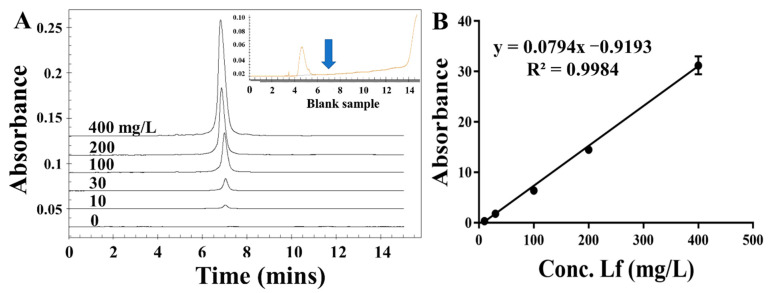
The electropherograms of different concentrations of LF standard (**A**) and the calibration curve (**B**).

To assess the applicability and accuracy of the ELAA kit, the calibration curve and sensitivity in the milk powder matrix were conducted. We chose one commercially available milk powder labeled with no Lf as the blank sample that was also confirmed by using the HPCE method (Figure 5 insert picture). The milk powder was subjected to a simple pre-treatment. After centrifugation, the intermediate layer of liquid was removed to be used as a matrix solution, by which the stocked solution of Lf standard was diluted to different concentrations ranging from 25 to 500 nM. Though assessed by the ELAA kit, Figure 4D showed that the relative A_450_ values in the microwells gradually became larger as the Lf concentration increased, and the calibration curve was obtained as y = 0.5299x − 0.6062 with the LOD of 17.08 nM (1.36 mg/kg). Furthermore, the R^2^ in matrixes was greater than 0.99, showing good linearity in the analytical range. Such high sensitivity could determine potential Lf content in milk powders. However, a slightly different slope between regression equations obtained by “sample matrix” and “standard solutions” indicates a matrix effect that can affect the results. Thus, the recovery experiment was also conducted using the matrix calibration curve by spiking the Lf standard into milk powder before pre-treatment to five distinct concentrations. Table 2 showed that the recovery rates of samples were varied in the range of 95–107%, as well as their RSD values of less than 2%. The results confirmed the reliability and accuracy of the ELAA kit to determine Lf sensitively in the milk powder matrix.

### 3.5. Application Evaluation in Real Samples

To evaluate the actual application of the ELAA kit, we determined the Lf content in thirteen samples that were purchased from the market and consisted of three kinds of dairy products, including goat milk powder (G1-G6), cow milk powder (C1-C6), nutrition drop (N-1), as well as with different Lf concentrations. Furthermore, these samples were also subjected to the verified assay by employing the HPCE method (Figure 5), and the CE results were regarded as the yardstick. Compared with the HPCE results (Table 3), the eleven results in the ELAA kit showed that the Lf concentrations are fundamentally consistent with HPCE values, along with the accuracy rates of these samples limited in the range of 79.71%~116.99%, as well as their RSD values of less than 3.61%. In particular, the blank samples are all tested accurately, indicating a good performance of the ELAA kit in real samples. There are two samples with slightly large differences in test results (138.34% and 146.43%), which may be due to their more complex matrix composition. However, the ELAA kit took only 60 min to accomplish analysis for all samples by only performing a single operation, which was much shorter than that required by the HPCE of about 12 h (including washing time) for all samples.

## 4. Conclusions

In this study, an enzyme-linked aptamer colorimetry kit was constructed to determine Lf in dairy products. To achieve a robust determination, the sequential reaction was employed between the recognition probes with the measured samples and subsequent capture probes, in which the indirect competition allowed full-coverage binding of the aptamers to Lf and avoided excessive binding. By a “mix-wash-detect” operation, the proposed ELAA kit demonstrates low limits of detection, good stability, and specificity under the optimized conditions, as well as a good calibration curve and high recovery rates in the spiked matrix. Furthermore, it also achieved Lf determination in eleven samples for three kinds of dairy products with different Lf concentrations, and the results were confirmed by the CE method with high accuracy. In the absence of rapid and accurate analysis for Lf determination in dairy products, our aptamer-based ELAA kit offers considerable advantages in terms of convenience, detection speed and time (~60 min), throughput (96-wells), and cost of analysis (~36 USD).

## Figures and Tables

**Table 1:** Reproducibility of the ELAA kit.

Conc. Lf (nM)	Intra-Day Assay			Inter-Day Assay			Intra-Batch Assay		
	Mean ^a^	SD ^b^	CV ^c^	Mean	SD	CV	Mean	SD	CV
60	1.34	0.09	0.07	1.37	0.01	0.01	1.11	0.04	0.03
80	1.04	0.01	0.01	1.16	0.04	0.03	0.92	0.03	0.03
100	0.80	0.05	0.06	0.82	0.01	0.02	0.67	0.01	0.02

^a^ Values represent the average of the A_450_ values of the parallel samples (*n* = 3). ^b^ Values represent the standard deviation of the parallel results (*n* = 3). ^c^ CV = SD/mean.

**Table 2:** Recovery tests in milk powder matrix using the ELAA kit.

Spiked (nM)	Found (nM)	Recovery (%)	RSD (%)
50	53.40	106.80	1.94
100	101.90	101.90	1.41
150	142.70	95.13	1.98

**Table 3:** Results of thirteen samples using the HPCE method and ELAA kit.

Sample	Label (nM)	HPCE (nM)	ELAA (nM)	Accuracy (%)	RSD (%)
C-1	0	0	0	100	0.03
C-2	0	0	0	100	0.39
C-3	420	351.5	403.1	114.70	1.41
C-4	480	521.1	499.1	95.78	2.26
C-5	600	594.6	599.3	100.78	0.73
C-6	360	220.2	304.6	138.34	2.81
G-1	0	0	0	100	0.43
G-2	0	0	0	100	0.23
G-3	540	572.1	469.5	82.07	2.82
G-4	510	402.5	381.6	94.81	0.13
G-5	78	122.3	179.1	146.43	0.05
G-6	600	288.7	337.7	116.99	0.03
N-1	9000	8961.8	7144.0	79.71	3.61

## Data Availability

All related data and methods are presented in this paper. Additional inquiries should be addressed to the corresponding author.

## Supplementary Materials

The following supporting information can be downloaded at: https://www.mdpi.com/article/10.3390/foods11233763/s1, Table S1: The comparison of ELISA and ELAA.
