# Supplementary material for: Enzyme-Linked Aptamer Kits for Rapid, Visual, and Sensitive Determination of Lactoferrin in Dairy Products
Source: Foods. 2022 Nov 22;11(23):3763. doi: 10.3390/foods11233763 (PMC9736959; doi:10.3390/foods11233763)
Supplement: Supplementary file 1 [file foods-11-03763-s001.zip › foods-1989830-supplementary.pdf]

Enzyme-linked aptamer kits for rapid, visual and sensitive determination of lactoferrin in dairy products

Fan Zhang [1,2], Hongxia Du [1,2], Linsen Li [3], Tengfei Li [4], Jing Wang [5], Zilei Chen [1,2], Mengmeng Yan [1,*], Chao Zhu [1,2,*] and Feng Qu [3]

[1] Institute of Quality Standard and Testing Technology for Agro-Products, Shandong Academy of Agricultural Sciences, Jinan 250100, China
[2] Shandong Provincial Key Laboratory Test Technology on Food Quality and Safety, Jinan, 250100, China
[3] Key Laboratory of Molecular Medicine and Biotherapy, School of Life Science, Beijing Institute of Technology, Beijing 100081, China
[4] School of Life Sciences and Food Engineering, Hebei University of Engineering, Handan 056038, China
[5] School of Materials Science and Engineering, Hebei University of Engineering, Handan 056038, China
* Correspondence: ynky202@163.com (M.Y.); ndytzhuchao@126.com (C.Z.)

Table S1. The comparison of ELISA and ELAA.

LOD (nM)	Detection strategy	Detection duration (h)	reference
1.25	Indirect competition(ELISA)	1	[26]
5.25	Direct Competition(ELISA)	1	[36]
40.38	Sandwich method(ELISA)	2.75	[37]
225	Direct Competition(ELISA)	2.5	[38]
14.01	Indirect competition(ELAA)	1	this work
